# Recurrent device-related thrombosis after left atrial appendage closure with the watchman FLX: A case report and literature review

**DOI:** 10.1016/j.ijcrp.2025.200527

**Published:** 2025-10-14

**Authors:** Yuemiao Jiao, Yue Yu, Guangyuan Song, Chengqian Yin

**Affiliations:** Department of Interventional Center of Valvular Heart Disease, Beijing Anzhen Hospital, Capital Medical University, Beijing, 100029, China

## Abstract

**Background:**

Left atrial appendage closure (LAAC) effectively lowers stroke risk in atrial-fibrillation (AF) patients who cannot tolerate long-term anticoagulation. Device-related thrombosis (DRT), although infrequent, carries a threefold increase in subsequent embolic events and remains a therapeutic challenge, even with the newer Watchman FLX occluder.

**Case summary:**

A 72-year-old woman with paroxysmal AF (CHA_2_DS_2_-VASc = 5; HAS-BLED = 2) underwent LAAC with a 30 mm Watchman FLX after bleeding-limited warfarin use. She was prescribed dual antiplatelet therapy (DAPT) post-procedure. Eight weeks later, cardiac CT detected a device-surface thrombus; warfarin (INR 2.5–3.0) achieved complete resolution by 7 months. Despite continued anticoagulation, repeat CT at 22 months revealed a larger thrombus. Transesophageal echocardiography confirmed recurrent DRT.

**Discussion:**

This case underscores multifactorial DRT pathogenesis: patient-specific hypercoagulability (age, persistent AF, PAI-1 variant), anatomic factors (large LAA, 30 mm device), and premature INR reduction. Current evidence indicates that early hypoattenuation thickening on cardiac CT, peri-device leak, and suboptimal antithrombotic regimens are associated with DRT. Emerging data support CT-based surveillance, individualized anticoagulation—potentially favoring direct oral anticoagulants (DOACs)—and next-generation, endothelialization-oriented device designs.

**Conclusion:**

Recurrent, large-burden DRT can occur late after Watchman FLX implantation despite initial thrombus resolution and guideline-directed therapy. Optimal management requires (1) vigilant, multimodality imaging follow-up; (2) stringent, patient-tailored anticoagulation with real-time INR or DOAC level assessment; (3) consideration of genetic or laboratory markers of thrombophilia; and (4) advances in device bioengineering to accelerate endothelial healing. Further studies should refine risk-stratified antithrombotic strategies and validate imaging biomarkers to pre-empt DRT in high-risk LAAC recipients.

## 1 Introduction

Left atrial appendage closure (LAAC) is an important interventional strategy for preventing atrial thrombus formation and embolic events in patients with non-valvular atrial fibrillation, particularly those at high bleeding risk [1]. However, device-related thrombosis (DRT) remains a significant complication, occurring in 3.8 % of cases and tripling the risk of post-procedural stroke [2]. The WATCHMAN FLX device has shown improved safety and efficacy compared to its predecessor, with a reported incidence of DRT of approximately 1.7 % [3]. We present a rare case of recurrent large thrombus formation following LAAC using the Watchman FLX device. This case highlights the challenge of the DRT-related treatment after LAAC.

## 2 Timeline

February 13, 2023: The patient underwent pre-procedural evaluation for left atrial appendage closure.

February 16, 2023: LAAC was successfully performed with implantation of a 30 mm Watchman FLX device.

April 10, 2023: A pulmonary vein CT scan identified thrombus formation in the left atrium, suspected to be device-related. The patient was initiated on warfarin therapy with a target INR of 2.0–3.0.

November 06, 2023: Follow-up transesophageal echocardiography (TEE) demonstrated complete resolution of the thrombus. Warfarin therapy was continued.

December 21, 2024: Repeat CT imaging revealed enlargement of the left atrial thrombus compared to the April scan, despite ongoing anticoagulation.

December 23, 2024: TEE confirmed the presence of recurrent thrombus, indicating refractory device-related thrombosis.

## 3 Case presentation

A 72-year-old woman with paroxysmal atrial fibrillation, first diagnosed in 2020, was evaluated for LAAC. The primary indication was the patient's intolerance to oral anticoagulation. She had experienced recurrent bruising and gingival bleeding while on warfarin, leading to discontinuation. Although these events were not life-threatening, and did not qualify as a major bleeding history, the patient expressed strong reluctance to resume long-term anticoagulation therapy despite counseling on its benefits and risks. After shared decision-making, LAAC was offered as an alternative stroke-prevention strategy. She reported intermittent palpitations and paroxysmal nocturnal dyspnea, consistent with New York Heart Association (NYHA) class Ⅲ symptoms. Her past medical history included hypertension of more than 10 years (Grade Ⅲ, well controlled with medication) and valvular disease characterized by severe tricuspid and mild-to-moderate mitral regurgitation. There was no history of ischemic stroke, coronary artery disease, diabetes mellitus, or chronic kidney disease. The patient was a lifelong nonsmoker and did not consume alcohol.

On physical examination, blood pressure was 138/82 mmHg, and the heart rate was irregularly irregular at 92 beats/min. Jugular venous distension and bilateral lower limb pitting edema were present. Pulmonary auscultation revealed bibasilar crackles. Cardiac auscultation demonstrated an irregularly irregular rhythm with a pansystolic murmur at the apex radiating to the axilla, and a holosystolic murmur at the left lower sternal border. Peripheral pulses were palpable and symmetric, with a pulse rate lower than the heart rate.

Her CHA_2_DS_2_-VASc score was 5, and her HAS-BLED score was 2. Laboratory evaluation revealed a serum creatinine level of 66.40 μmol/L with an estimated glomerular filtration rate (eGFR) of 66.4 mL/min/1.73 m^2^. The B-type natriuretic peptide (BNP) level was elevated at 440.0 pg/mL. Genetic screening for thrombophilia identified a heterozygous 5G/4G polymorphism in the plasminogen activator inhibitor-1 (PAI-1) gene, while Factor V Leiden testing was negative (wild-type GG genotype).

**Fig. 1:**
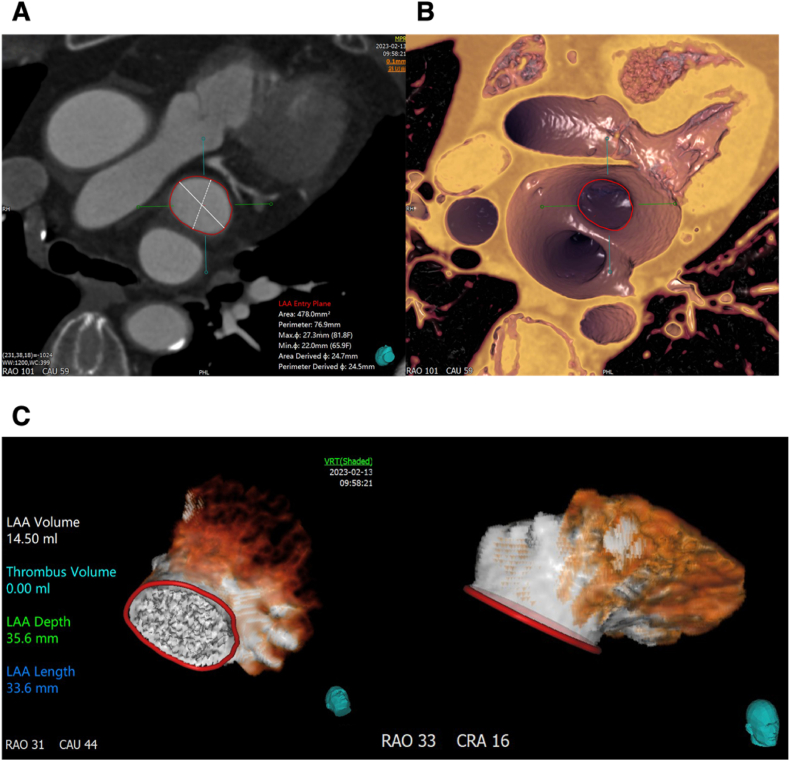
**Pre-procedural Multidetector Computed Tomography Assessment of the Left Atrial Appendage** (A, B) Multiplanar reconstructions show ostial area of 478 mm^2^ (maximum diameter 27 mm, minimum 22 mm). (C) Three dimensional volume rendered images (right anterior oblique 31°, caudal 44°) demonstrate an cauliflower shaped LAA volume of 14.5 mL, depth 35.6 mm, and length 33.6 mm.

**Fig. 2:**
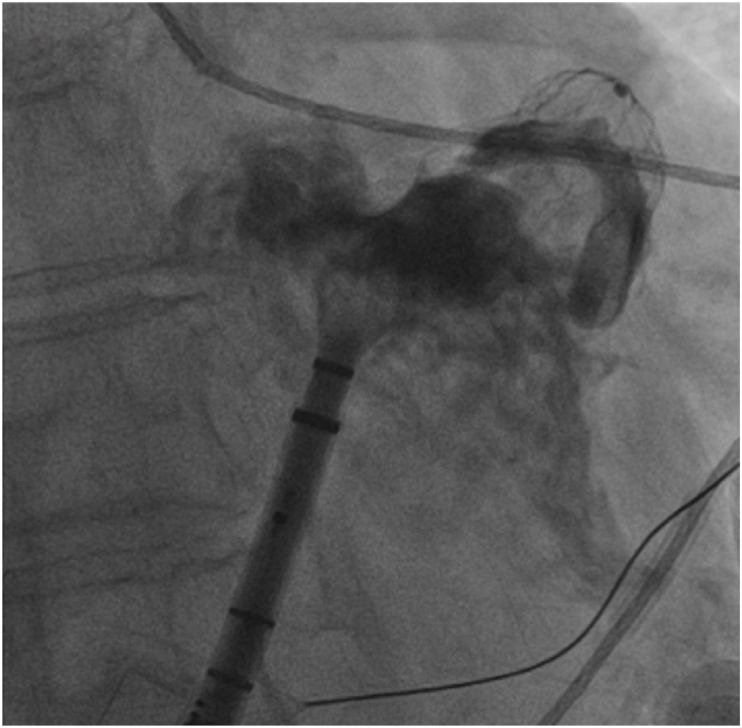
Fluoroscopic angiography during watchman FLX Deployment.

Baseline transthoracic echocardiography showed biatrial enlargement, severe tricuspid regurgitation, and mild-to-moderate mitral regurgitation. The multidisciplinary heart team formulated a stepwise treatment plan: considering the patient's history of atrial fibrillation and the high bleeding risk associated with long-term anticoagulation, the initial strategy was to perform left atrial appendage closure (LAAC) to simplify postoperative anticoagulation management and reduce thromboembolic complications, followed by re-evaluation for possible tricuspid valve surgery. Pre-procedural cardiac CT, performed on February 13, 2023 (Fig. 1), revealed a cauliflower-shaped left atrial appendage with a maximum orifice diameter of 27 mm. Based on CT and intraoperative angiographic measurements, LAAC was performed in April 2023 using a 30 mm Watchman FLX device. Intraprocedural angiography outlined the LAA and confirmed coaxial alignment of the 30 mm Watchman FLX device before release, with contrast washout indicating complete device apposition and peri-device leak (PDL) < 5 mm (Fig. 2). Intraprocedural transesophageal echocardiography (TEE) confirmed appropriate device positioning and adequate sealing, with residual peri-device flow measuring less than 5 mm, meeting the criteria for successful implantation.

Intra procedural angiography outlines the LAA and confirms coaxial alignment of the 30 mm Watchman FLX device before release, with contrast washout indicating complete device apposition and minimal peri-device leak (PDL).

**Fig. 3:**
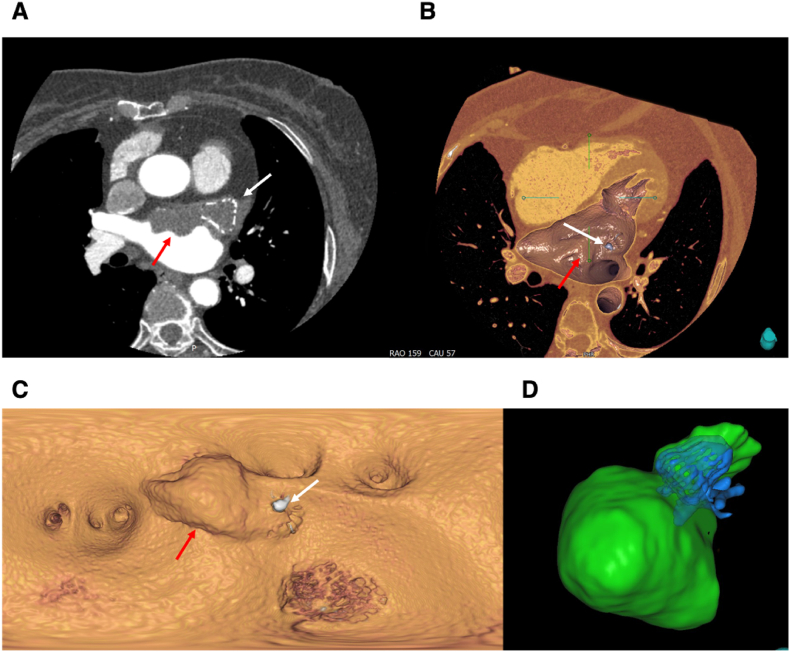
**First Follow up Computed Tomography Fifty Days After Implantation Demonstrating Device Related Thrombosis** (A) Axial cross-sectional CT image showing the Watchman FLX occlude (white arrow) and adjacent atrial surface thrombus (red arrow). (B) Three-dimensional volume-rendered reconstruction highlighting the occluder (white arrow) and overlying thrombus (red arrow). (C) Virtual endoscopic view revealing a hypoattenuating layered thrombus (red arrows) adherent to the atrial surface of the device (white arrow), consistent with early DRT. (D) Volume-rendered reconstruction demonstrating the Watchman FLX well seated within the LAA without displacement or significant peridevice leak, indicating that thrombus formation occurred despite optimal device placement. (For interpretation of the references to colour in this figure legend, the reader is referred to the Web version of this article.)

**Fig. 4:**
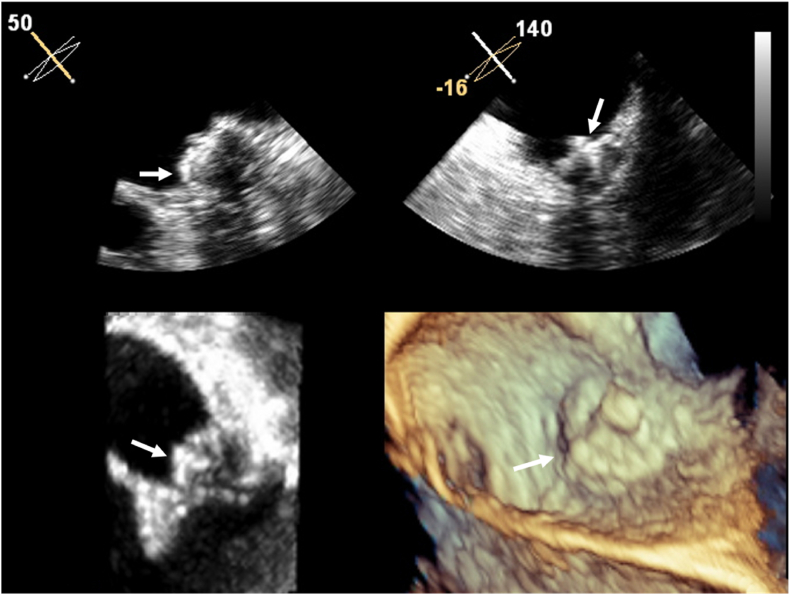
Transesophageal echocardiography seven months post implantation showing thrombus resolution.

According to the WATCHMAN FLX instructions for use, post-implantation antithrombotic regimens include (A) short-term oral anticoagulation plus aspirin, or (B) dual antiplatelet therapy (DAPT) for 45 days followed by single antiplatelet therapy. Given that her prior bruising and gingival bleeding did not constitute a contraindication to anticoagulation, regimen (A)—oral anticoagulation plus aspirin—would have been guideline-directed. However, considering the patient's strong reluctance to restart anticoagulation, a compromise strategy of DAPT (aspirin 100 mg and clopidogrel 75 mg daily) was selected. During postoperative management, the patient received optimal medical therapy (OMT) focused on standard heart failure management and internal milieu stabilization. The regimen included a β-blocker (metoprolol), an angiotensin receptor–neprilysin inhibitor (ARNI, sacubitril/valsartan), an SGLT2 inhibitor (dapagliflozin), diuretics (torasemide and spironolactone), and a potassium supplement (Panangin). Throughout the follow-up period, these heart failure medications remained largely stable; however, the antithrombotic regimen required multiple adjustments due to the subsequent occurrence of device-related thrombosis (DRT). At 8 weeks post-implantation, a CT scan revealed a device-related thrombus (DRT) adherent to the occluder surface (Fig. 3). Therapy was consequently switched from DAPT to warfarin (1.125 mg/day; target INR 2.5–3.0). A follow-up TEE in November 2023 (Fig. 4) showed complete thrombus resolution, indicating effective anticoagulation. The patient was advised to continue warfarin therapy and maintain regular INR monitoring. In November 2023, the patient developed moderate normocytic normochromic anemia with a hemoglobin decline (Hb 88 g/L), elevated serum creatinine (113.1 μmol/L), positive fecal occult blood testing, and no abnormalities in urinalysis, with INR 3.12. These abnormalities subsequently resolved within 1 month. The transient changes were most likely attributable to occult gastrointestinal bleeding with volume depletion, leading to reversible prerenal azotemia. Although warfarin-related nephropathy (WRN) could not be completely excluded, the absence of hematuria and full recovery of renal function rendered this explanation less probable.

**Fig. 5:**
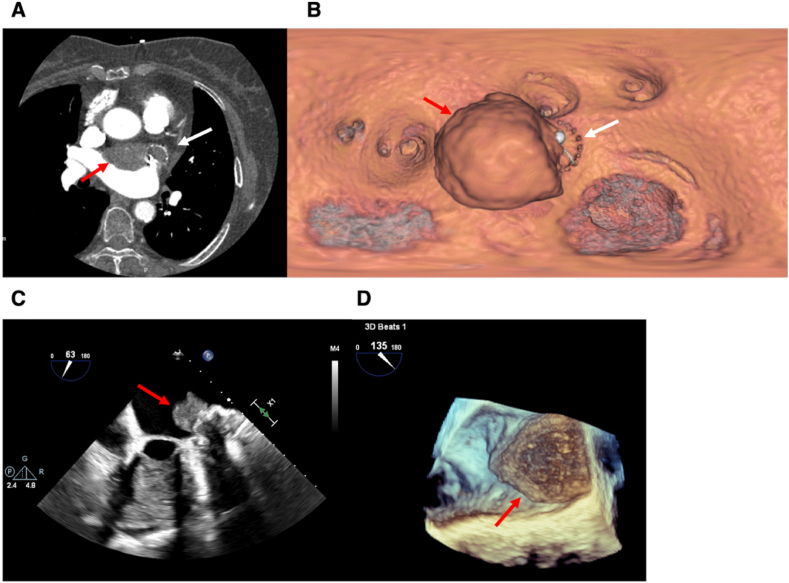
**Recurrent Device-related Thrombosis Eight Months After Watchman FLX Implantation.** (A) Multidetector CT (December 21, 2023) shows a new thrombus (red arrow) on the atrial surface of the Watchman FLX occluder (white arrow). (B) Virtual endoscopic view illustrates the device (white arrow) with an overlying thrombus (red arrow). (C) Two-dimensional transesophageal echocardiography confirms the recurrent thrombus (red arrow). (D) Three-dimensional transesophageal echocardiography demonstrates the thrombus (red arrow) adherent to the device surface. (For interpretation of the references to colour in this figure legend, the reader is referred to the Web version of this article.)

However, over the course of long-term oral anticoagulation therapy, the patient exhibited poor adherence to warfarin therapy and irregular INR monitoring, which led to suboptimal anticoagulation control. She also experienced intermittent episodes of melena during this period, further complicating anticoagulation management. On December 21, 2024, during follow-up at our center, her INR was INR 1.68. A subsequent CT scan on the same day (Fig. 5A and B), revealed a recurrent—and possibly enlarged—thrombus on the device. Upon discovery of the thrombus, anticoagulation was immediately switched from warfarin to low-molecular-weight heparin (LMWH). TEE on December 23, 2024 (Fig. 5C), confirmed recurrent DRT, indicating a refractory course despite an initially favorable response to anticoagulation. Complementary cardiac CT not only identified the second thrombus but also, through three-dimensional reconstruction, revealed incomplete endothelialization of the device surface. These findings underscore the pathophysiological role of delayed endothelial healing in DRT formation and highlight the clinical challenge of maintaining adequate anticoagulation in patients with high bleeding risk and poor adherence to therapy. Follow-up CT and TEE examinations revealed no peri-device leak. Importantly, no ischemic stroke occurred during the post-procedural follow-up period.

Multiplane transesophageal echocardiography demonstrates complete resolution of the previously noted thrombus on the device surface (white arrow) following initiation of warfarin, showing a smooth atrial-facing surface of the occluder and no residual peri-device flow.

## 4 Discussion

Our case underscores the multifactorial nature of DRT, integrating clinical, genetic, and procedural determinants, and highlights the importance of individualized antithrombotic strategies and multimodality surveillance.

1. Independent risk factors for DRT

This patient presents with multiple prothrombotic factors: advanced age, persistent atrial fibrillation, heart failure with biatrial enlargement, peripheral vascular disease, and significant valvular lesions—which collectively promote blood stasis, delay endothelialization, and increase thrombosis risk [[4], [5], [6], [7]]. [[4], [5], [6], [7]] The high CHA_2_DS_2_-VASc score, originally intended to estimate stroke risk in atrial fibrillation, has demonstrated predictive value for DRT, indicating some overlap between systemic and device-specific thrombogenicity [8]. Implantation of a large (30 mm) device in a cauliflower-shaped appendage may further delay endothelialization and exacerbate local stasis [[9], [10], [11]].

Laboratory and genetic markers provide additional refinement of risk: elevated D-dimer, markers of heightened thrombin generation, and prothrombotic genotypes (e.g., plasminogen activator inhibitor-1 variants) have been implicated in impaired fibrinolysis and abnormal clot formation. enriched for subsequent DRT [12,13]. Additional modifiable risks included suboptimal anticoagulation adherence and labile INR.

Temporally, DRT is typically detected at a median of 93 days post-procedure [14], with cases reported as early as immediately after implantation [15] and as late as 4 years [16]. Early DRT relates to incomplete endothelialization and a transient hypercoagulable state, marked by elevated thrombin, fibrinogen, and von Willebrand factor up to 45 days post-procedure [12,17]. By contrast, late DRT likely reflects delayed or aberrant endothelialization and/or persistent inflammation that sustain a chronically thrombogenic device–blood interface [[18], [19], [20]].

2. Antithrombotic strategies in patients at high bleeding risk

Post-procedural antithrombotic therapy (ATT) reintroduces significant bleeding hazards during the endothelialization period. Early trials established the original regimen of Warfarin plus aspirin for 45 days, followed by dual antiplatelet therapy (DAPT) for 6 months, then aspirin alone [[21], [22], [23]]. A network meta-analysis compared various ATT strategies and found DOAC monotherapy to be superior in reducing both thromboembolic events and major bleeding, with significantly lower all-cause mortality versus with VKA (OR: 0.39; 95 % CI: 0.17–0.89) [24]. Still, no randomized trial has defined the optimal regimen, and therapy must be individualized, suggesting that individualized adjustment is needed for anticoagulation therapy after LAAC [25]. Importantly, dose reductions outside of guideline-specified criteria (age, weight, renal function) may compromise efficacy [26]. Apixaban has demonstrated a more favorable bleeding profile than warfarin and select DOACs across a broad range of renal function—including eGFR <15 mL/min. Contemporary evidence supports apixaban as one of the preferred anticoagulant options for CKD, particularly in patients at high bleeding risk [27]. Furthermore, apixaban has been documented to facilitate resolution of left ventricular thrombus, suggesting broader therapeutic potential in high-risk populations [28]. This suggests its potential therapeutic value in selected high-risk populations. Integrating this evidence with the present case, characterized by fluctuating renal function and a history of bleeding, a Direct Oral Anticoagulant (DOAC)—specifically apixaban—may be considered a more suitable alternative for future management.

3. Optimization of internal environment and valve intervention

Beyond antithrombotic therapy, systemic optimization is essential. Active management of biomarkers—including CRP, lipid profile, blood glucose, renal function, and INR—supports thrombotic risk reduction, with Optimal Medical Therapy (OMT) stabilizing inflammatory and metabolic status [29]. OMT also establishes the foundation for evaluating suitability for structural interventions such as Transcatheter Tricuspid Valve Replacement (TTVR).

TTVR has been shown to improve symptoms and quality of life in patients with complex anatomy unsuitable for repair, with the TRISCEND II trial demonstrating superior outcomes versus OMT alone [30,31]. These findings underscore the clinical value of transcatheter tricuspid valve intervention as a promising therapeutic option for carefully selected patients with advanced valvular disease.

4. Future directions: combinatorial strategies and device innovation

Complex clinical scenarios such as recurrent device-related thrombosis after left atrial appendage closure highlight the need for multidimensional and combinatorial strategies that transcend traditional single-modality approaches. Future management should integrate optimized pharmacotherapy, systemic metabolic and inflammatory modulation, advanced imaging surveillance, genetic and hemodynamic profiling, and, when indicated, structural reintervention. The emerging discipline of combinatorial biomedicine provides a conceptual and methodological framework for such integrative care, emphasizing the synergistic application of biomedical engineering, artificial intelligence, and clinical medicine to achieve individualized precision therapy [32]. In parallel, continued device innovation—particularly surface modifications to accelerate endothelialization and reduce thrombogenicity—offers promise for safer long-term outcomes [[33], [34], [35]].

### 4.1 Study limitations

A limitation in this case is the lack of pulmonary venous CTA assessment following the initiation of intensified anticoagulation therapy. Due to the patient's advanced age, cardiac dysfunction, and borderline elevated creatinine levels, the examination was not performed. Consequently, the presence of any residual shunt remains uncertain.

## 5 Conclusion

Recurrent, large burden DRT can occur late after Watchman FLX implantation despite initial thrombus resolution and guideline directed therapy. In patients with high thrombotic risk and limited anticoagulation tolerance, management should emphasize: (1) vigilant, multimodality imaging follow up; (2) individualized Pharmacological strategy; (3) risk stratification incorporating clinical, anatomical, and genetic markers; and (4) advances in device bioengineering to accelerate endothelial healing. These insights support refinement of antithrombotic protocols and tailored surveillance to preempt DRT in vulnerable LAAC recipients.

## CRediT authorship contribution statement

**Yuemiao Jiao:** Writing – original draft, Investigation, Data curation, Conceptualization. **Yue Yu:** Visualization, Resources, Data curation. **Guangyuan Song:** Writing – review & editing, Supervision, Formal analysis. **Chengqian Yin:** Writing – review & editing, Supervision, Resources, Project administration, Conceptualization.

## Funding

This work was financially supported by the grant from the 10.13039/501100012166National Key R&D Program of China (2022YFC3600201).
